# Surfactant replacement therapy for respiratory distress syndrome in preterm infants: United Kingdom national consensus

**DOI:** 10.1038/s41390-019-0344-5

**Published:** 2019-02-19

**Authors:** Sujoy Banerjee, Ramon Fernandez, Grenville F. Fox, Kevin C. W. Goss, Helen Mactier, Peter Reynolds, David G. Sweet, Charles C. Roehr

**Affiliations:** 1 Singleton Hospital, Swansea, UK; 2 Royal Sussex County Hospital, Brighton, UK; 3 Evelina London Children’s Hospital, London, UK; 4 Leeds Centre for Newborn Care, Leeds Children’s Hospital, Leeds, UK; 5 Princess Royal Maternity, Glasgow, UK; 6 St. Peter’s Hospital, Ashford and St. Peter’s Hospitals NHS Foundation Trust, Chertsey, UK; 7 Royal Jubilee Maternity Hospital, Belfast, UK; 8 Medical Sciences Division, University Department of Paediatrics, University of Oxford, Oxford, UK; 9 Newborn Services, John Radcliffe Hospital, Oxford University Hospitals, NHS Foundation Trust, Oxford, UK

## Introduction

Our aim was to develop consensus recommendations from United Kingdom (UK) neonatal specialists on the use of surfactant for the management of respiratory distress syndrome RDS in preterm infants. RDS due to surfactant deficiency is common in preterm infants. Signs, including tachypnoea, recessions, and grunting, usually commence shortly after birth, and increase in severity during the first 12–48 h of postnatal life. Significant RDS may require mechanical ventilation (MV) or noninvasive ventilatory support (NIV), both of which have potential to cause lung injury via a number of mechanisms.^1^ The aim of RDS management is to provide appropriate respiratory support whilst minimising complications and, ultimately, bronchopulmonary dysplasia (BPD). Treatment with exogenous surfactant reduces requirement for positive pressure ventilation, mitigates risk of pulmonary air leak, and improves survival.^1^

International consensus guidelines on management of RDS have been published;^1^ however, recent developments in the field of less invasive surfactant administration prompt the need for a UK national consensus on surfactant use in preterm infants with, or at risk of, RDS.

## Methods

For the purpose of this expert consensus, UK specialists in neonatal respiratory disease and its management were selected based on their previous clinical and scientific experience of RDS management. In a three-step modified Delphi process, seven recommendations were debated and modified in a series of iterative reviews, with the goal of reaching an expert panel consensus. The final stage of the Delphi process was a face-to-face meeting to discuss the precise wording and hierarchy of the recommendations. The Delphi process was supported by an unconditional grant from Chiesi UK Limited; however, the authors maintain complete control over all content of the recommendations.

## Key recommendations

Consensus recommendations for the management of RDS in preterm infants were developed; this manuscript provides a narrative for these recommendations. The recommendations are as follows:

**1. All neonatal units should have an agreed policy for the management of early RDS**.

Minimising unwarranted variation in care is an NHS (National Health Service) mandate (www.england.nhs.uk/rightcare; www.1000livesplus.wales.nhs.uk), and European Standards of Care for Newborn Health (www.newborn-health-standards.org) require that guidelines for management of RDS should be available in individual units.

**2. Early rescue surfactant rather than prophylaxis is recommended. In some situations, this may include surfactant administration in the delivery suite**.

Early rescue involves surfactant treatment for RDS early in the course of the disease when additional oxygen requirement is still relatively low, usually within a few hours after birth. Surfactant prophylaxis is traditionally defined as surfactant administration solely on the basis of gestational age and/or expected high risk of RDS. In the modern context of noninvasive ventilatory management, surfactant prophylaxis and unnecessary intubation has potential for harm. A systematic review of studies comparing surfactant administration through intubation as prophylaxis versus stabilisation on continuous positive airway pressure (CPAP) with early rescue surfactant (if required) showed that infants initiated on CPAP were at lower risk of chronic lung disease or death.^2^ The potential benefit or harm of prophylactic surfactant administration using less invasive surfactant administration (LISA) techniques is at present unknown.

Whilst advocating avoidance of routine prophylaxis, it is important to recognize that in some situations, surfactant may need to be administered very soon after birth. This would include infants who require intubation for stabilisation soon after birth, or who have rapidly increasing oxygen requirements beyond initial stabilisation. Surfactant should therefore always be available in the delivery suite, and this should be acknowledged in local policies for early RDS management.

**3. In babies with evolving RDS, rescue surfactant should be administered early in the course of the disease. Inspired oxygen concentration above 30% in the first hours of life is a reasonable predictor of CPAP failure**.

The incidence of CPAP failure, defined as the need for MV following initiation of CPAP, increases with lower gestational age at birth.^3^ Infants who fail initial CPAP therapy have an increased incidence of adverse outcomes including mortality, pneumothorax, intraventricular hemorrhage, and BPD, compared with infants for whom CPAP is successful.^3^ In infants with evolving RDS, rescue surfactant should be used in an effort to prevent CPAP failure.^3^ The inspired oxygen concentration required to achieve acceptable oxygen saturations as measured by pulse oximetry is widely available to clinicians as a proxy to assess the severity and progression of RDS. According to Dargaville et al., inspired oxygen concentration above 30% for preterm infants in the first hours of life is strongly predictive of CPAP failure, and is typically reached more rapidly by infants with lower gestational ages.^3^ Although the above mentioned study was concerned with identifying predictors of CPAP failure, the group of experts believes that this oxygen threshold may be considered appropriate for NIV generally. Inspired oxygen concentration should also be interpreted in combination with clinical assessment of work of breathing, direction of improvement or worsening, and the general clinical condition of the infant.

**4.(a)For rescue therapy using natural surfactants, poractant alfa at an initial dose of 200** **mg/kg reduces mortality and the need for redosing compared to 100** **mg/kg of surfactant**.^4^ **There is an absence of data on other natural surfactants at doses** **>100** **mg/kg**.

This recommendation is based on currently available evidence regarding natural surfactants and their dosing via an endotracheal tube for the treatment of RDS in preterm infants. Natural surfactants vary in their origin, composition, and recommended dosing, and studies have shown differences in clinical effect. Poractant alfa (Curosurf^®^, Chiesi Farmaceutici S.p.A., Parma, Italy) and beractant (Survanta^®^, AbbVie Inc., North Chicago, USA) are the only surfactants currently licensed for the use in the UK. An initial dose of 200 mg/kg of poractant alfa appears more effective than 100 mg/kg of poractant alfa or beractant in reducing the need for repeat dosing.^4^ Currently, poractant alfa is the only surfactant that is licensed at a 200 mg/kg initial dose. There are no comparative dosing data for surfactants administered using less invasive techniques.

**4.(b)The surfactant dose should be calculated and administered based on the baby’s weight. If surfactant is required before the baby’s birth weight is known, it is reasonable to use whole vial dosing based on an estimated weight**.

This recommendation supports accurate dosing of surfactant, which should be calculated based on the infant's weight. Weight-based dosing applies to both early rescue administration and treatment of established RDS. For some surfactant preparations of lesser volume (e.g. poractant alfa), whole-vial dosing, based on estimated weight, is reasonable, for example when administered for early rescue in the delivery suite.

**4.(c)A second and sometimes a third dose of surfactant can be considered in ongoing RDS**.

On occasions, it may be necessary to administer a second, and sometimes a third dose of surfactant in ongoing RDS (lack of improvement, persistent high oxygen requirement, increased work of breathing on noninvasive support, or continuing need for MV).^1^ Other causes of respiratory failure, which may require different treatments, should be considered. Clinical trials comparing single versus multiple doses of surfactant administered via endotracheal intubation showed better outcomes if multiple doses were allowed.^1^ Subsequent dosage should be guided by the manufacturer’s maximum cumulative dosage recommendations.

**5. There is emerging evidence that the LISA technique may be the preferred method for spontaneously breathing preterm infants kept on NIV, as an alternative to the intubation-surfactant-extubation (INSURE) technique**.

INSURE describes surfactant administration after endotracheal intubation, followed by brief ventilation, timely extubation (within 60 min) and reinstitution of NIV.^1^ However, the implementation of INSURE is variable, particularly with regard to the length of MV following surfactant administration; in some instances, MV may be prolonged for different reasons, including physician’s preference. The LISA technique, also referred to as minimally invasive surfactant therapy (MIST), is a method for administering surfactant via an endotracheally placed catheter to infants spontaneously breathing on NIV.^1^ A soft-tipped, semi-rigid, fine bore surfactant administration catheter is placed under direct laryngoscopy, with or without the use of Magill forceps; equipment specifically designed for this use is available. Surfactant is then given slowly whilst the infant continues to breathe. Vital signs are continuously monitored and patient comfort ensured throughout the procedure, during which NIV is uninterrupted. Moderate desaturation, with or without bradycardia, may occur, in which case surfactant administration should pause without removal of the catheter. The catheter is removed once the surfactant delivery is complete.

Current evidence from several randomised controlled trials of variable quality synthesised in meta-analyses shows that in preterm infants on NIV, the LISA procedure reduces need for MV and risk of death and BPD, compared with endotracheal intubation, surfactant application, and MV.^5^ The neonatal workforce applying LISA should be appropriately trained. Up-to-date education on theory, mannequin simulations, and the use of video laryngoscopy is advisable. Clear guidelines and local audits should be in place to identify local strengths and challenges for LISA implementation.
